# Function of aquaporins in sepsis: a systematic review

**DOI:** 10.1186/s13578-018-0211-9

**Published:** 2018-02-09

**Authors:** Katharina Rump, Michael Adamzik

**Affiliations:** Klinik für Anästhesiologie, Intensivmedizin und Schmerztherapie, Universitätsklinikum Knappschaftskrankenhaus Bochum-Langendreer, Ruhr-Universität Bochum, In der Schornau 23-25, 45882 Bochum, Germany

**Keywords:** Aquaporin, AQP, Expression, Immune cells, Migration, Brain, Kidney, Liver, Lung, Heart, LPS, sepsis

## Abstract

**Background:**

Sepsis is a common cause of death in intensive care units worldwide. Due to the high complexity of this immunological syndrome development of novel therapeutic strategies is urgent. Promising drug targets or biomarkers may depict aquaporins (AQPs) as they regulate crucial key mechanisms of sepsis.

**Main body:**

Here we report on base of the current literature that several AQPs are involved in different physiological processes of sepsis. In immune system mainly AQPs 3, 5 and 9 seem to be important, as they regulate the migration of different immune cells. Several studies showed that AQP3 is essential for T cell function and macrophage migration and that AQP5 and AQP9 regulate neutrophil cell migration and impact sepsis survival. Additionally, to the function in immune system AQPs 1 and 5 play a role in sepsis induced lung injury and their downregulation after inflammatory stimuli impair lung injury. By contrast, AQP4 expression is up-regulated during brain inflammation and aggravates brain edema in sepsis. In kidney AQP2 expression is downregulated during sepsis and can cause renal failure. Some studies also suggest a role of AQP1 in cardiac function.

**Conclusion:**

In conclusion, AQPs are involved in many physiological dysfunctions in sepsis and their expressions are differently regulated. Additional research on the regulatory mechanisms of aquaporins may identify potential therapeutic targets.

## Background

Sepsis is one of the most common complications in Intensive Care Units in Germany and the United States [1, 2], and mortality remains unrestrainable high due to the extreme complexity of this immunological syndrome. Predictive biomarkers which characterize this immunological syndrome properly are still missing; hence no individual therapy adapted on the immune status of the unique patient can be conducted. Aquaporins might be convenient biomarkers because they play an important role in inflammation and especially in sepsis as revealed by experimental and association studies [3–6].

Aquaporins (AQPs) are a group of to date 13 identified membrane proteins, which are essential for the regulation of water and salt in- and out flux of the cell. In addition, some AQPs facilitate the passive transport of glycerol and other small solutes such as urea and carbon dioxide through the cell membrane [7]. The water-selective AQPs are involved in many biological functions, including transepithelial fluid transport, cell migration, brain edema and neuroexcitation [7], whereas the aquaglyceroporins participate in cell proliferation, adipocyte metabolism and epidermal water retention. With this study we want to elucidate the possible role of AQPs in pathomechanisms of sepsis on base of the current literature.

### Approach of literature research and methodology

**Fig. 1:**
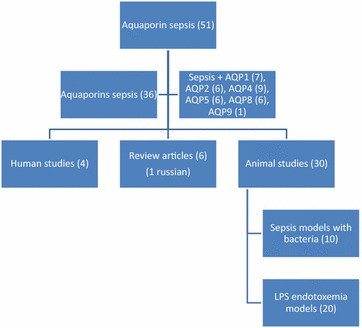
Workflow of literature research

A literature search was undertaken using various online sources of English journal articles including ScienceDirect, PubMed and Web of Science. The keywords “aquaporin AND sepsis”, “aquaporins AND sepsis” and “AQP(xy) AND sepsis” were used to search all relevant articles dealing with the role of aquaporins in sepsis. In total 51 studies were found. 10 articles were excluded because they either did not deal with sepsis or with aquaporins. One article was excluded because it was in Russian. The workflow of literature research can be found in Fig. 1. Due to the relative low number of articles dealing with real bacterial sepsis models, endotoxemia models using LPS injection were included in the analysis.

### Aquaporin expression during inflammation

To completely understand the role of AQPs in sepsis, it is important to know how their expression is altered during inflammation. It was demonstrated that in leucocytes of septic patients *AQP3* expression is reduced 2.5 [8] fold and that simultaneously *AQP1* expression is increased twofold [8]. In line with this our group showed that *AQP1* expression is increased in the monocytic cell line THP-1 after lipopolysaccharide (LPS) administration, but *AQP5* mRNA expression is reduced [9]. *AQP6* expression in contrast might play a role in viral infections as it is decreased after viral infection and in turn can reduce the infectivity of Hazara virus [10]. Furthermore, *AQP8* is reduced in hepatocytes after LPS administration [11]. In addition, patients with systemic inflammatory response syndrome (SIRS) show increased *AQP9* expression in neutrophils compared to healthy controls [12]. Moreover, Gram-negative bacteria as *P. aeruginosa* induce increased expression, distribution and re-organization of *AQP9* in macrophages with is accompanied by changes in macrophage size and morphology. This in turn affects motility, migration and phagocytosis [13].

### Aquaporins in cell migration of immune cells

**Fig. 2:**
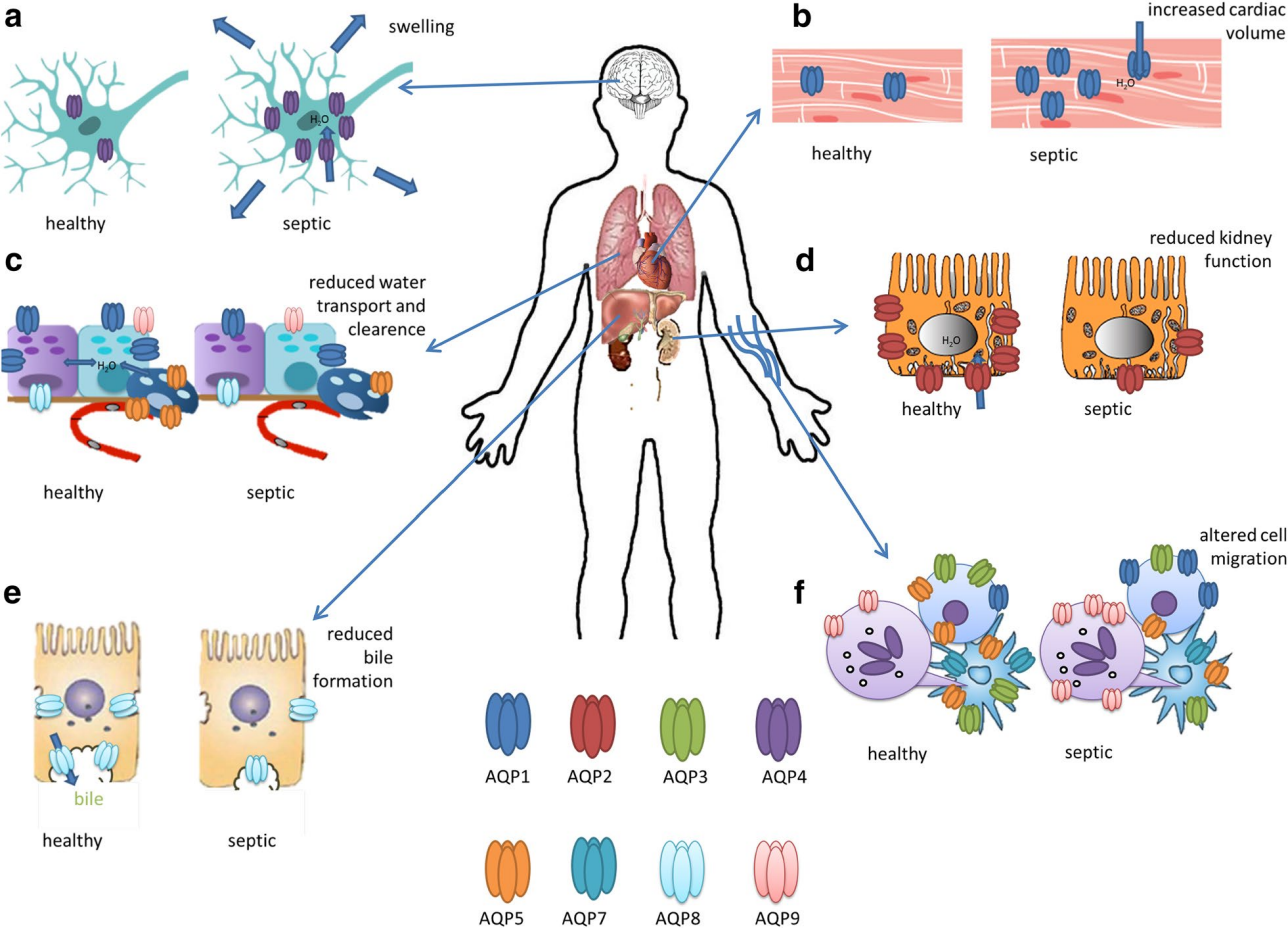
Purposed roles and expressions of aquaporins in sepsis: **a** AQP4 is expressed in brain and increased in sepsis, **b** AQP1 expression is increased in cardiac cells in sepsis, **c** AQP1, AQP8, AQP9 are expressed in bronchiolar epithelial cells and AQP5 can be found in alveolar epithelial cells; their expressions are reduced in sepsis, **d** AQP2 appears in the apical and subapical part of collecting duct principal cells and is reduced in sepsis, **e** AQP8 is reduced in hepatocytes in sepsis, **f** AQP1 and AQP9 expressions are increased in neutrophils and lymphocytes in sepsis, whereas the expression of AQP3, AQP5 and AQP7 is reduced in lymphocytes and dendritic cells (Figure modified and adapted from [70–74, 77–79])

The importance of aquaporins in cell migration has been demonstrated several times before [7, 14–17]. The proposed mechanism by which AQPs enhance cell migration is that they facilitate water influx at the cell’s leading edge. This causes membrane expansion and formation of a concentration gradient of actin polymers which is followed by actin repolymerization to stabilize the membrane protrusion and lamellipodia formation [17]. As immune cell migration is an essential mechanism in sepsis, AQPs might depict key players in this process. Considerable AQPs for immune cell migration are AQP1, AQP3, AQP5, AQP7 and AQP9 as they are expressed in activated B and T lymphocytes (AQP1, 3, 5) [15] as well as immature dendritic cells (AQP3, 5, 7) [15] and neutrophils (AQP9) [15, 18, 19] (Fig. 2f).

AQP5 seems to be of special interest, because in the past our group demonstrated that the C-allele of the functional *AQP5* A(-1364)C promoter polymorphism (rs3759129) is associated with increased survival in severe sepsis [3] but decreased *AQP5* expression [20]. Recently we showed that *Aqp5*-knockout (KO) mice show increased survival compared to wildtype mice after LPS induced endotoxemia. Furthermore, AQP5 overexpression caused increased migration of the T-lymphocytic cell line Jurkat. In addition, neutrophil granulocytes from C-allele carriers showed decreased migration compared to A-allele carriers. Therefore we concluded that the *AQP5* genotype and AQP5 protein expression seem to alter neutrophil cell migration and may influence survival in sepsis by altering neutrophil cell migration. Hence AQP5 might be a key protein in inflammation and depict a novel target for developing sepsis therapeutics [21].

Similar to our study Zhu et al. analyzed the effects of *Aqp3* expression in a sepsis mouse model. They found that mouse resident peritoneal macrophages (mRPMs) express the aquaglyceroporin *Aqp3* and to a low extent *Aqp7* and *Aqp9* in a plasma membrane pattern [22]. In contrast to our study, *Aqp3*-KO mice show significantly greater mortality than wildtype mice in a model of bacterial peritonitis. In addition, *Aqp3*-KO is accompanied by reduced migration of macrophages [22]. Besides to macrophage function, AQP3 seems also to be crucial for T-cell migration. It is suggested that AQP3-mediated H_2_O_2_ uptake is required for chemokine-dependent T-cell migration and a sufficient immune response [23].

AQP4 plays a role in the development of regulatory T-cells in the thymus. *Aqp4*-KO mice show decreased levels of CD4+ CD25+ regulatory T-cells. The decreased amount of regulatory T-cells causes increased microglial inflammatory response in a mouse model of Parkinson with *Aqp4*-KO mice [24].

Similar to the role of AQP5 and AQP3, AQP9 seems to be responsible for neutrophil migration, as *Aqp9*-KO mice show reduced neutrophil migration to fMLP [25]. In addition, *Aqp7*-KO mice have reduced migration of cutaneous dendritic cells. Beside its role in cell migration AQP7 seems to be responsible for antigen uptake as it could be demonstrated that *Aqp7*-deficient DCs showed a decreased cellular uptake of low-molecular-mass compounds and high-molecular-mass substances [19].

### Role of aquaporins in the inflammasome

The inflammasome is an important key modulator of the immune response and affects the immune response by the release of proinflammatory cytokines. It can be found in macrophages and neutrophil granulocytes and can recognize pathogens like bacteria. The inflammasome inter alia consists of NLR family pyrin domain containing 3 (NLRP3) which is up-regulated in sepsis [26]. Activation of NLRP3 inflammasome causes interleukin 1 beta (IL-1β) release. The IL-1β release depends on the pH of the cell and its regulation is caused by water influx mediated by aquaporins. AQP-mediated water movement in macrophages therefore appears as the common element unifying the variety of NLRP3 inflammasome activators [27].

### Aquaporins in sepsis induced brain inflammation

One devastating complication of sepsis is septic encephalopathy (SE) [28]. In this context, aquaporins might play an important role, as SE is associated with vasogenic brain edema [29, 30]. The inflammation of the brain occurring in SE is mediated by neutrophil infiltration and causes *Aqp4* upregulation which aggravates brain edema [31, 32] (Fig. 2a). Upregulation of *Aqp4* in brain after LPS exposure can be attenuated by dexamethasone and this mechanism is mainly regulated by tumornecrosis factor alpha (TNF-α) [33]. However the use of corticosteroids like dexamethasone in sepsis is still discussed and its usage is only recommended under certain conditions [34].

In addition, AQP4 expression is upregulated in astrocytes during sepsis induced delirium (SID) and exosomes carrying AQP4 proteins from astrocytes to the peripheral blood may be utilized as biomarker for SID [35].

### Aquaporins in kidney injury

Another common complication in sepsis is acute kidney injury (AKI), former called acute renal failure (ARF), which is frequently associated with polyuria and urine concentration defects and it increases the mortality rate in sepsis [36]. A cecal ligation and puncture (CLP) mouse model for sepsis showed that *Aqp2* expression is downregulated through NF-κB pathway and may therefore cause acute renal failure during sepsis [37] (Fig. 2d). Pretreatment of rats with continuous erythropoietin receptor activator (CERA) preserves *Aqp2* expression in rat kidneys and protects against sepsis induced AKI [38].

The downregulation of *Aqp2* in sepsis models is confirmed by animal models using LPS induced endotoxemia after short time exposure (6 h) [39–42], whereas after long time exposure (18 h) *Aqp2* expression is increased in kidney [43]. Another study shows that *Aqp2* is downregulated after LPS administration in an LPS sepsis model in rats [44] and that pretreatment but not post-treatment with propofol prevents *Aqp2* downregulation and protects renal function during endotoxemia and that this effect may be mediated by regulation of Intercellular Adhesion Molecule 1 (ICAM-1), TNF-α and mediators of apoptosis [44]. Another possibility for *Aqp2* preservation after LPS exposure is treatment with α-lipoic acid [45].

### Aquaporins in liver dysfunctions during sepsis

Liver has numerous functions in sepsis and is itself a target for sepsis induced injury [46]. For example septic shock and its toxins can cause hypoxic hepatitis, cholestasis due to altered bile metabolism or hepatocellular and acute liver injury [46]. In cholestasis AQP8 might play a role as it is downregulated after LPS stimulation in hepatocytes via TNF-α [11, 47]. The reduced AQP8 expression in turn causes reduced water permeability of hepatocytes, which can result in reduced bile formation and aggravates cholestasis [48, 49] (Fig. 2e). Beside, AQP8 can modulate hepatocellular mitochondria function by modifying water transport [50]. A loss of mitochondria function in turn can cause kidney injury due to loss of cellular energy [51]. In an endotoxemia rat model hepatic mitochondrial *Aqp8* expression is reduced [52]. Regulation of *Aqp8* in endotoxemia and septic models by substances like tetramethylpyrazine or ethyl pyruvate could stabilize the mitochondria membrane potential, protect hepatocellular mitochondria from damage and might therefore be a therapeutic option in sepsis [51, 53].

### Aquaporins in cardiac dysfunction

40–50% of patients with prolonged septic shock develop cardiac dysfunction [54] and newer studies indicate that cardiac dysfunction can occur in all stages of sepsis [55]. The underlying molecular mechanisms are not fully understood yet, but a notable cause is mitochondrial dysfunction which contributes to cardiac dysfunction by causing myocardial energy depletion [56]. Here AQP1 might be important because *Aqp1* knockout causes cardiac hypertrophy in mice [57] (Fig. 2b). Another animal study tested the hypothesis if Aqp1 may play a role in cardiac dysfunction during sepsis. They found that *Aqp1* expression is increased after LPS exposure in cardiac tissue and that this might influence cardiac function [58].

### Aquaporins in acute lung injury

Another common complication in sepsis is acute lung injury that can cause acute respiratory distress syndrome (ARDS), which is associated with increased risk of in-hospital mortality [59]. In lung mainly the aquaporins AQP1 and 5, 8 and to a lower extent AQP9 are expressed [60]. Here, Aqp1 is expressed in all vascular endothelial cells, Aqp5 in the alveolar type I cells and Aqp8 and Aqp9 can be found in the bronchial epithelial cells in lung [61] (Fig. 2c). In 2016 in a small group of septic patients suffering from diffuse alveolar damage is was demonstrated that they have increased expression of AQP3 and AQP5 in the alveolar septum compared to healthy controls [62]. Recently it was demonstrated that *Aqp5* expression is decreased after sepsis induction with cecal ligation puncture (CLP) in the lung of rats [63, 64]. This effect can be attenuated by emodin [65] and is regulated by the microRNAs miR-96 and miR-330 [66]. In line with this *Aqp1* expression is decreased after LPS exposure in rat lungs [6, 67]. As a therapeutic option it was demonstrated that hydrogen rich saline and parenteral vitamin C can be protective in sepsis related lung injury and that it can attenuate the LPS induced reduction of *Aqp1* and *Aqp5* expression [5, 68]. In addition, *Aqp1* and *Aqp5* expression in lung is reduced in lung after an inflammatory pancreatitis models, whereas *Aqp8* and *Aqp9* expression remains unaffected [61]. Here the traditional Chinese prescription Dai-Huang-Fu-Zi-Tang can upregulate *Aqp1* and 5 and attenuate inflammation [61].

## Conclusion

The regulatory mechanisms of aquaporins by LPS after endotoxemia and in sepsis seem to be tissue and aquaporin specific, as it can be seen in Table 1 and Fig. 2. As an example and it was demonstrated that AQP8 is downregulated in hepatic cells after LPS administration, though TNF-α pathway [11], while AQP9 expression remains unaffected [33, 69].

**Table 1:** Overview of AQP regulation during inflammation (↑ upregulation, ↓ downregulation, ? unknown regulation, = unaffected)

Aquaporin	Tissue	Regulation during inflammation	References
*AQP1*	Immune cells	↑ In leukocytes and cell lines (THP-1)	[8, 9]
	Heart	↑ In cardiac cells	[58]
	Lung	↓ In lung tissue after LPS	[5, 6]
*AQP2*	Kidney	↓ In renal tissue after LPS	[37]
*AQP3*	Immune cells	↓ In leukocytes of septic patients	[8]
*AQP4*	Brain	↑ In brain and anterior pituitary gland	[31, 75]
*AQP5*	Lung	↓ In lung tissue after LPS	[65]
	Immune cells	↓ In THP-1 cells after LPS	[9]
*AQP7*	Immune cells	? Mouse resident peritoneal macrophages	[76]
*AQP8*	Liver	↓ In hepatic cells	[11]
	Lung	= In bronchial epithelial cells	[61]
*AQP9*	Immune cells	↑ In neutrophils of SIRS patients	[12]
	Immune cells	? Mouse resident peritoneal macrophages	[76]
	Lung	= In bronchial epithelial cells	[61]

In summary, AQPs protein expressions seem to alter differential pathological mechanisms in sepsis and might be key proteins in inflammation. As a limitation of this review it has to be mentioned that several results were concluded from animal studies and that they potentially might to be fully adopted to human physiology. Elucidating the differential regulatory mechanisms of AQP expression in human studies might be helpful for developing novel sepsis therapeutics.

## Abbreviations

AQP: aquaporin
CLP: cecal ligation and puncture
KO: knockout
LPS: lipopolysaccharide
SE: septic encephalopathy
TNF: tumor necrosis factor
WT: wildtype
